# The Effects of Eyestalk Ablation on the Androgenic Gland and the Male Reproductive Organs in the Kuruma Prawn *Marsupenaeus japonicus*

**DOI:** 10.3390/ani15243556

**Published:** 2025-12-11

**Authors:** Takehiro Furukawa, Fumihiro Yamane, Takuji Okumura, Taeko Miyazaki, Naoaki Tsutsui

**Affiliations:** 1 Department of Life Sciences, Graduate School of Bioresources, Mie University, 1577 Kurimamachiyacho, Tsu 514-8507, Mie, Japantaeko@bio.mie-u.ac.jp (T.M.); 2 Mie Prefectural Fish Farming Center, 3564-1 Hamajima, Hamajimacho, Shima 517-0404, Mie, Japan; 3 Fisheries Technology Institute, Japan Fisheries Research and Education Agency, 422-1 Nakatsuhamaura, Minamiise 516-0193, Mie, Japan; okumura_takuji42@fra.go.jp

**Keywords:** decapod crustacean, testis, vas deferens, seminal vesicle, IAG

## Abstract

Insulin-like androgenic gland factor (IAG) is considered a key regulator of male sexual differentiation and maturation in decapod crustaceans. In several species, *IAG* expression is thought to be negatively regulated by the eyestalk, as demonstrated by eyestalk ablation (ESA) experiments. In the kuruma prawn *Marsupenaeus japonicus*, however, the upstream regulatory mechanisms of *IAG* (*Maj-IAG*) remain largely unclear. In the present study, males of different body sizes were subjected to ESA to elucidate these mechanisms. Bilateral ESA induced upregulation of *Maj-IAG* expression from day 7 onward, whereas unilateral ESA did not. Moreover, enhanced development of male reproductive organs and hypertrophy of the androgenic gland were observed from day 7 after bilateral ESA. These findings indicate that *Maj-IAG* is regulated by eyestalk-derived factor(s), supporting the presence of an eyestalk–androgenic gland endocrine axis in *M. japonicus*. By contrast, the expression of *Maj-Dsx2*, a homolog of doublesex (*Dsx*) that has recently been proposed as an upstream regulator of IAG, did not show a consistent increase following bilateral ESA across all experiments, suggesting that the involvement of *Maj-Dsx2* in this axis remains unclear. Overall, this study provides fundamental insights into the regulatory mechanisms of decapod male reproduction.

## Simple Summary

In decapod crustaceans, insulin-like androgenic gland factor (IAG) is produced by the male-specific androgenic gland. IAG is regarded as the crustacean androgen and is thought to be regulated by eyestalk-derived factor(s). In this study, we performed eyestalk removal in individuals of three different body sizes and assessed changes in gene expression and the size of male reproductive organs to investigate the regulatory mechanisms of IAG in the kuruma prawn *Marsupenaeus japonicus*, an important aquaculture species. Bilateral eyestalk removal resulted in the upregulation of *IAG* gene expression in seminal vesicle containing the androgenic gland and increased relative weights of testis, vas deferens, and seminal vesicle to body weight, suggesting that IAG plays a critical role in development and maintenance of male reproductive functions. In contrast, unilateral eyestalk removal did not induce upregulation of *IAG* expression, suggesting that a complex regulatory mechanism might underlie the control of IAG expression in *M. japonicus*, and that the unilateral response in this species may differ from those reported in other decapods. These findings provide fundamental insights into the regulatory mechanisms of IAG and contribute to the establishment of the molecular basis of reproductive control in decapod crustaceans.

## 1. Introduction

Following its initial discovery in the amphipod *Orchestia cavimana* [1], subsequent studies have established the androgenic gland (AG) as a pivotal endocrine organ controlling the expression of male secondary sexual characteristics in malacostracans. In the isopod *Armadillidium vulgare*, AG implantation into early-stage females induced abnormal differentiation of ovaries into testes, accompanied by the development of male secondary sexual characteristics [2]. The androgenic gland hormone (AGH) was first characterized in *A. vulgare* as the AG-specific hormone responsible for these sexual changes [3]. In decapod species, feminization was induced by AG ablation in males of the giant freshwater prawn *Macrobrachium rosenbergii* [4]. Conversely, masculinization was achieved by AG implantation into females of the Australian red claw crayfish *Cherax quadricarinatus* [5] and *M. rosenbergii* [6], and even functional sex reversal was achieved by AG implantation into females of *M. rosenbergii* [7]. The identity of the putative AGH-like molecule inferred from these findings was first elucidated through molecular characterization in *C. quadricarinatus* [8]; it was designated as insulin-like AG factor (IAG), based on its primary structural features. Subsequently, homologous genes were characterized in several other decapod species. Functional analyses of IAG have mainly been conducted in freshwater decapod species, revealing that IAG is involved in the regulation of sexual differentiation, similar to the action of AGH [9,10,11,12]. Therefore, IAG has been referred as a key regulator of male sexual differentiation and maturation in decapod crustaceans [13].

The eyestalk is known to play a pivotal regulatory role upstream of *IAG*. Eyestalk ablation (ESA) leads to an upregulation of *IAG* expression in several species, including the blue crab *Callinectes sapidus*, the oriental river prawn *Macrobrachium nipponense*, the pacific white shrimp *Litopenaeus vannamei*, and the banana shrimp *Fenneropenaeus merguiensis* [14,15,16,17]. Hypertrophy of the AG has also been reported in other species as one of the morphological changes following ESA [18,19,20]. Moreover, endocrine activity of the hypertrophied AG following ESA has been suggested to be enhanced in the Harris mud crab *Rhithropanopeus harrisii* [21]. The X-organ/sinus gland (XO-SG) complex, located within the eyestalk ganglia, is responsible for the synthesis and release of multiple neuropeptides. These peptides are involved in diverse physiological processes, including hemolymph glucose control, molting, and vitellogenesis in females. Accordingly, the corresponding molecules, crustacean hyperglycemic hormone (CHH) regulating glucose homeostasis, molt-inhibiting hormone controlling molting, and vitellogenesis-inhibiting hormone regulating reproduction, were identified as members of the CHH family [22]. In addition, CHH-family peptides have been suggested to participate in the regulation of *IAG* expression [23,24]. Moreover, crustacean female sex hormone from the XO-SG has been reported to inhibit the development of AG in the mud crab *Scylla paramamosain* [25]. Although further research is needed to determine whether common factors are involved, these findings strongly suggest the presence of an inhibitory regulatory mechanism originating from the XO–SG [19,22,26]. This mechanism, in turn, supports the eyestalk–AG–testis endocrine axis proposed by Khalaila et al. [19], as a key regulatory pathway in male reproduction of decapod crustaceans.

Recently, doublesex (*Dsx*), a polymorphic transcription factor belonging to the DMRT family, has been suggested to regulate *IAG* transcription in decapods. It was reported that *E. sinensis Dsx* acts as an upstream regulatory gene of *EsIAG* and contributes to male sex differentiation [27]. In the black tiger shrimp *Penaeus monodon*, *Dsx* is involved in male reproductive development and positively regulates *IAG* expression through specific binding to its upstream promoter region [28]. Similarly, in the Chinese shrimp *Fenneropenaeus chinensis*, *Dsx* is suggested to participate in sex differentiation by regulating *IAG* expression [29]. Taken together, *Dsx* may function as an upstream transcriptional regulator of IAG within the eyestalk–AG axis in decapod crustaceans.

The kuruma prawn *Marsupenaeus japonicus* is an important species for both fisheries and aquaculture in Japan. In this species, the AG of is attached to the distal region of the seminal vesicle (SV), and *Maj-IAG*, a homolog of IAG, was identified through full-length cDNA characterization [30]. Recently, our group reported that *Maj-IAG* regulates the development of secondary sexual characteristics in juvenile males [31]; however, the mechanism regulating its expression remains poorly understood. Moreover, two *Dsx* genes (*Maj-Dsx1* and *Maj-Dsx2*) have been identified in this species [32], but their involvement in the regulation of *Maj-IAG* expression is still unknown. Therefore, this study aimed to elucidate the regulatory mechanisms of *Maj-IAG* by evaluating the effects of ESA at molecular, morphological, and histological levels in male *M. japonicus*. We present the first integrated analysis of the effects of ESA on *Maj-IAG* expression together with AG vesicle area and male reproductive organ indices across different growth stages within this species. This approach provides novel insights into both the conservation of eyestalk–AG axis among decapod crustaceans and into possible interspecific differences in how eyestalk-derived inhibitory inputs contribute to the regulation of IAG expression.

## 2. Materials and Methods

### 2.1. Experimental Design

In this study, three experiments were conducted using sexually mature male kuruma prawns (*M. japonicus*) of three different sizes, all of which had already initiated spermatogenesis (Figure S1). Rearing and dissection of the prawns were conducted in accordance with the guidelines of Mie University and the Japanese policy for animal experimentation (https://www.scj.go.jp/ja/info/kohyo/pdf/kohyo-20-k16-2e.pdf (accessed on 24 November 2025)). Ethical approval was not required for experiments involving crustaceans under current regulations. In Experiment 1 and 2, the prawns were obtained from a local aquaculture farm (Naruto, Japan) and used in November 2020 and January 2024, respectively. In Experiment 3, the prawns were provided by Northern Ise Bay Intermediate Rearing Facility (Suzuka, Japan) and used in November 2021. All prawns were transferred to the Mie Prefectural Fish Farming Center (Shima, Japan) and reared in partitioned net cages (900 × 500 × 550 mm; width × depth × height, Tetron Raschel T-280, Tanaka Sanjiro Co., Ltd., Ogori, Japan) installed within tanks that were continuously supplied with sand-filtered natural seawater, which typically had a salinity of approximately 34 PSU, pH 8.0–8.1, and a dissolved oxygen concentration of around 6 mg/L. Aeration was also provided in each net cage by means of an air stone to ensure adequate oxygenation.

Each experiment was conducted after sufficient acclimation under the same rearing conditions. Prawns were fed a commercial diet (Goldprawn, Higashimaru Co., Ltd., Hioki, Japan) daily to satiation as a common condition. In Experiment 1, bilateral ESA was performed by cauterizing the eyestalks with heated forceps. Three net cages were installed in a 2-ton FRP tank covered with opaque light-shielding sheets (High Metallic Silver 0.1 mm, C.I. TAKIRON Corporation, Tokyo, Japan). Because prawns subjected to bilateral ESA are generally known to exhibit strong cannibalistic behavior, two net cages containing 15 prawns each were assigned to the ESA group, whereas one net cage containing 24 prawns was assigned to the control group. Sampling was scheduled for days 0, 4, 7, and 14, with day 0 defined as the day of ESA treatment. In Experiment 2, six net cages were installed in an outdoor 6-ton reinforced concrete tank covered with a light-shielding nets (Daio Raschel 2200, Innovex Co., Ltd., Tokyo, Japan), with three net cages assigned to the ESA group and three to control group. Each net cage contained 12–13 prawns. Sampling was scheduled for days 0, 14, and 22. In Experiment 3, bilateral and unilateral ESA were carried out, and four net cages were installed in a 2-ton FRP tank covered with opaque light-shielding sheets. Two net cages containing 12–13 prawns were assigned to the bilateral eyestalk-ablated group, whereas one net cage was assigned to each of the control and unilateral ESA groups, containing 19 and 25 prawns, respectively. Sampling was scheduled for days 0, 7, and 14. In all experiments, intermediate samplings were performed by evenly collecting prawns from each net within a treatment group so that the remaining number of prawns did not differ markedly among nets. Owing to differences in the number and body size of prawns used, and the available rearing space, the number of prawns placed in each net varied among experiments. However, as no significant difference in growth was observed among groups (Figure S2), this variation was considered to have no effect on the objectives of the present study. The detailed conditions are summarized in Table 1.

### 2.2. Sample Collection

Prawns from each group were dissected, excluding individuals immediately before or after molting. The reproductive organs were excised and separated into the testis, vas deferens (VD), and SV. Then, each organ was weighed by an electronic scale. For gene expression analysis, half of each tissue sample was preserved in RNA*later* solution (Thermo Fisher Scientific, Inc., Waltham, MA, USA) at −20 °C until RNA extraction. The remaining halves were fixed in Davidson’s or Bouin’s solution for 24 h and stored in 70% ethanol for histology.

### 2.3. Biometric Indices

Male reproductive organ indices, including the testis somatic index (TSI; testis weight/BW × 100), the VD somatic index (VDSI; VD weight/BW × 100), and the SV somatic index (SVSI; SV weight/BW × 100), were calculated.

### 2.4. Total RNA Extraction, Reverse Transcription, and Quantitative Real-Time PCR

The primers and TaqMan probe used for quantitative real-time PCR (RT-qPCR) (Table 2) were designed based on sequences deposited in GenBank for *Maj-IAG* (AB598415), *doublesex 1* (*Maj-Dsx1*, LC569703), and *doublesex 2* (*Maj-Dsx2*, LC569704) [32,33]. Total RNA was extracted from one SV of each individual using a NucleoSpin RNA isolation kit (Macherey–Nagel GmbH & Co. KG, Düren, Germany) according to the manufacturer’s instructions, and RNA concentration was measured with a NanoDrop Lite spectrophotometer (Thermo Fisher Scientific). All RT-qPCR reactions were run on a 7300 Real-Time PCR System (Applied Biosystems, LLC, Foster City, CA, USA).

In Experiment 1, first-strand cDNA was synthesized from 500 ng of total RNA using the High Capacity cDNA Reverse Transcription Kit (Applied Biosystems) with random primers in a 20 µL reaction volume, under the following conditions: 25 °C for 10 min, 37 °C for 120 min, and 85 °C for 5 min. The resulting cDNA was diluted fourfold with 10 mM Tris–HCl (pH 8.0). *Maj-IAG* expression was quantified using a probe-based assay with Luna Universal Probe qPCR Master Mix (New England Biolabs, Ipswich, MA, USA), whereas *Maj-Dsx1* and *Maj-Dsx2* were quantified using an intercalating-dye assay with Luna Universal qPCR Master Mix (New England Biolabs). Each 14 µL reaction contained 1 µL of cDNA (corresponding to 6.25 ng total RNA), 400 nM of each primer, and 200 nM of TaqMan probe (for *Maj-IAG* only). Thermal cycling conditions included 95 °C for 1 min, followed by 40 cycles of 95 °C for 15 s and 60 °C for 60 s.

In Experiments 2 and 3, one-step RT-qPCR was used. *Maj-IAG* expression was analyzed using the Luna Universal Probe One-Step RT-qPCR Kit (New England Biolabs), and *Maj-Dsx1* and *Maj-Dsx2* were analyzed using the Luna Universal One-Step RT-qPCR Kit (New England Biolabs). Each 14 µL reaction contained 4 ng of total RNA, 400 nM of each primer, and 200 nM of TaqMan probe (for *Maj-IAG* only). Thermal cycling conditions were 55 °C for 10 min (reverse transcription), 95 °C for 1 min, followed by 45 cycles of 95 °C for 15 s and 60 °C for 60 s. All reactions were performed in duplicate. Relative gene expression levels were calculated on the basis of equal input nucleic acid per reaction (equal cDNA input for two-step RT-qPCR and equal total RNA input for one-step RT-qPCR), without normalization to a reference gene.

### 2.5. Histology of the Male Reproductive Organs

The tissues of testis, VD, and SV were dehydrated in ethanol series (70%, 80%, 90%, 95% and 99.5%) as appropriate. After embedding in paraffin wax, serial sections of 5–7 µm thickness were prepared using the RM2125RT microtome (Leica Microsystems GmbH, Wetzlar, Germany). Because the AG is attached to the SV, the SV was sectioned transversely from the pore side. Since it is not possible to identify in advance which sections contained the AG, many sections were used. Ensuring that the sections were placed through the same region of the AG was technically difficult. After rehydration, the sections were stained with hematoxylin and eosin, dehydrated, and mounted using MGK-S mounting medium (Matsunami Glass Ind., Ltd., kishiwada, Japan). Sections were observed under the Axio Imager.A1 light microscope (Carl Zeiss Microscopy GmbH, Jena, Germany), and digital images were captured using the Axiocam MR5 camera (Carl Zeiss) or the Axiocam 208 color camera (Carl Zeiss) mounted on the microscope.

### 2.6. Measurement of AG Area

The area of AG vesicles was measured using Fiji/ImageJ software version 1.54f [34,35] on captured digital images. For each individual, the areas of vesicles with clearly distinguishable boundaries and containing multiple AG cells were measured. In one field of view under a 40× objective lens, the areas of as many vesicles as possible were measured. If fewer than 10 vesicles per individual could be measured in a field, vesicles in an additional field of view were also used. The mean area of vesicles (10–40) was calculated for each individual. This mean value was used as the representative AG vesicle area for that individual. Because it is technically challenging to ensure that all sections pass through the same anatomical region of the AG, some degree of unavoidable bias may remain in the measurements.

### 2.7. Statistics

All results were expressed as means with standard error of the means (SEM). Biological sample sizes (*n*) varied among analyses and experiments, ranging from 3–10 in Experiment 1, 4–26 in Experiment 2, and 5–7 in Experiment 3. The exact *n* values for each analysis are given in the corresponding figure legends. Statistical analysis was performed in R (version 4.1.2; R Core Team, 2023) with RStudio (version 2021.09.2+382; Posit, PBC, Boston, MA, USA). For two-group comparisons, the Wilcoxon rank-sum test was conducted using the exact method. For three-group comparisons, the Steel–Dwass test was applied using the asymptotic method, as the exact method was computationally impractical. The Jonckheere–Terpstra test (two-sided) for assessment of the trend in a group were conducted in cases where time-course data were available. Statistical significance was set at *p* < 0.05. Statistical analysis of RT-qPCR data was performed based on expression levels.

## 3. Results

### 3.1. Effects of ESA on Somatic Growth and the Development of Male Reproductive Organ

**Figure 1:**
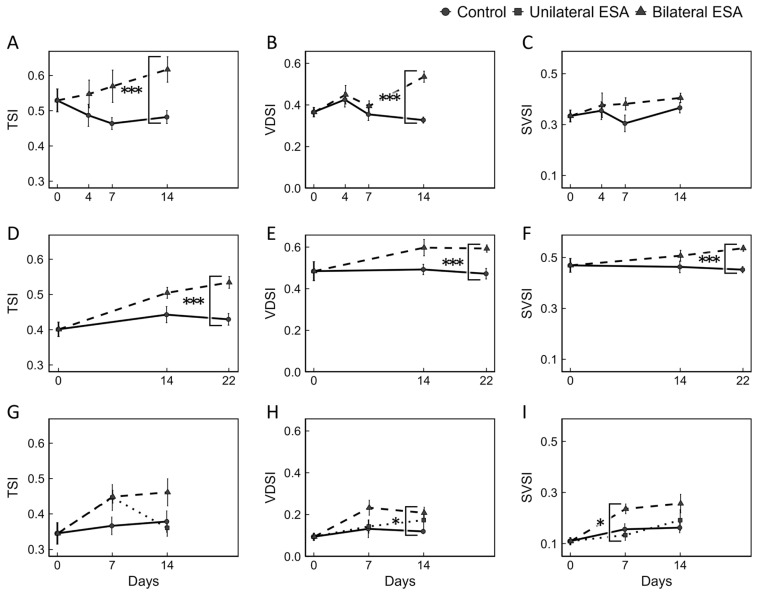
Effects of bilateral and unilateral ESA on the development of male reproductive organs. Changes in TSI (**A**,**D**,**G**), VDSI (**B**,**E**,**H**), and SVSI (**C**,**F**,**I**) are shown as mean ± SEM. Sample sizes for each group at each time point were as follows: (**A**–**C**) Experiment 1 control group, *n* = 10, 5, 7, and 7 on days 0, 4, 7, and 14, respectively, and bilateral ESA group, *n* = 5, 7, and 9 on days 4, 7, and 14, respectively; (**D**–**F**) Experiment 2 control group, *n* = 10, 12, and 16 on days 0, 7, and 14, respectively, and bilateral ESA group, *n* = 15 and 26 on days 7 and 14, respectively; (**G**–**I**) Experiment 3 control group, *n* = 6, 5, and 5 on days 0, 7, and 14, respectively, and unilateral ESA group, *n* = 7 and 7 on days 7 and 14, respectively, and bilateral ESA group, *n* = 5 and 5 on days 7 and 14, respectively. In all experiments, control values at day 0 were used as the baseline for each parameter. Dashed lines with triangles, dotted lines with squares, and solid lines with circles indicate the bilateral ESA, unilateral ESA, and control groups, respectively. Asterisks represent significant differences on the same day, as determined by Wilcoxon’s rank-sum test or Steel-Dwass test (* *p* < 0.05, *** *p* < 0.001).

In this study, the effects of ESA on growth and development of male reproductive organs were examined in individuals of three different sizes (Figure S1). Based on changes in BW, neither bilateral nor unilateral ESA affected somatic growth in all experiments (Figure S2A–C). In contrast, the development of male reproductive organs was promoted by bilateral ESA. In Experiment 1, TSI and VDSI on day 14 were significantly higher in the bilateral ESA group than in the control group (Figure 1A,B). The Jonckheere–Terpstra test revealed significant monotonic increasing trends in TSI, VDSI, and SVSI of bilateral ESA group over the experimental period. In Experiment 2, TSI, VDSI, and SVSI on day 22 were significantly higher in the bilateral ESA group than in the control group (Figure 1D–F). Significant monotonic increasing trends were also observed in TSI, VDSI, and SVSI of the bilateral ESA group during the experimental period.

Similar increasing trends were evident over time, as in Experiment 1. In Experiment 3, unilateral ESA was conducted in addition to bilateral ESA. Significant differences were observed in VDSI on day 14 between the bilateral ESA and control groups, and in SVSI on day 7 between bilateral ESA and unilateral ESA groups (Figure 1G–I). Significant monotonic increasing trends were detected in TSI and VDSI of the bilateral ESA group, and in SVSI of both the bilateral ESA and control groups during the experimental period. The increasing trends of the three parameters in the bilateral ESA group of Experiment 3 were consistent with those observed in Experiments 1 and 2.

### 3.2. Effects of ESA on Relative Expression Levels of Maj-IAG, Maj-Dsx1, and Maj-Dsx2 in SV

**Figure 2:**
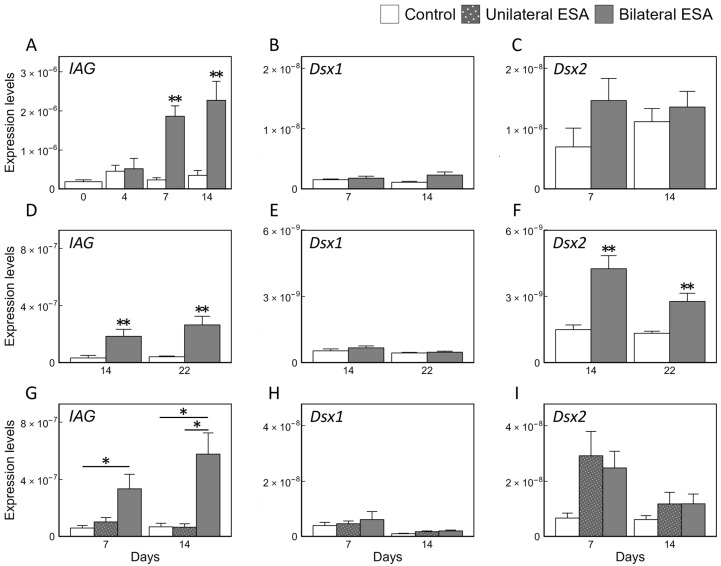
Effects of bilateral and unilateral ESA on gene expression levels. Relative expression levels of *Maj-IAG* (**A**,**D**,**G**), *Maj-Dsx1* (**B**,**E**,**H**), and *Maj-Dsx2* (**C**,**F**,**I**) in the SV, including the AG, were shown. Panels (**A**–**I**) present data from Experiments 1, 2, and 3, respectively. Bars show as mean + SEM. Sample sizes were as follows: (**A**) control group, *n* = 10, 5, 5, and 5 on days 0, 4, 7, and 14, respectively; bilateral ESA group, *n* = 5, 5, and 5 on days 4, 7, and 14, respectively; (**B**,**C**) both groups, *n* = 5 on days 7 and 14, respectively; (**D**) control group, *n* = 5 on days 14 and 22, respectively; bilateral ESA group, *n* = 6 and 7 on days 14 and 22, respectively; (**E**) control group, *n* = 5 and 4 on days 14 and 22, respectively; bilateral ESA group, *n* = 5 and 7 on days 14 and 22, respectively; (**F**) control group, *n* = 5 and 5 on days 14 and 22, respectively; bilateral ESA group, *n* = 6 and 7 on days 14 and 22, respectively; (**G**–**I**) both groups, *n* = 5 on days 7 and 14, respectively. Asterisks indicate significant differences on the same day, as determined by Wilcoxon’s rank-sum test or Steel-Dwass test (* *p* < 0.05, ** *p* < 0.01).

In Experiment 1, *Maj-IAG* expression levels were significantly higher in the bilateral ESA group than in the control group on days 7 and 14 (Figure 2A). No significant differences in the expression of either *Maj-Dsx1* or *Maj-Dsx2* were observed between the groups on days 7 and 14 (Figure 2B,C). In Experiment 2, *Maj-IAG* expression levels were again significantly higher in the bilateral ESA group than in the control group on days 14 and 22 (Figure 2D). No significant differences in *Maj-Dsx1* on days 7 and 14 (Figure 2E), whereas expressions of *Maj-Dsx2* on either day were also significantly higher in the bilateral ESA group than in the control group (Figure 2F). In Experiment 3, *Maj-IAG* expression levels in the bilateral ESA group were significantly higher than in the control group on day 7, and higher than in both the control and unilateral ESA groups on day 14 (Figure 2G). No significant differences in *Maj-Dsx1* and *Maj-Dsx2* on days 7 and 14 were detected among the groups (Figure 2H,I). Across all three experiments, bilateral ESA consistently induced an increase in *Maj-IAG* expression. The expression levels of *Maj-Dsx2* were basically higher than those of *Maj-Dsx1* in Experiments 2 and 3.

### 3.3. ESA Effects on AG Area and Spermatogenesis

**Figure 3:**
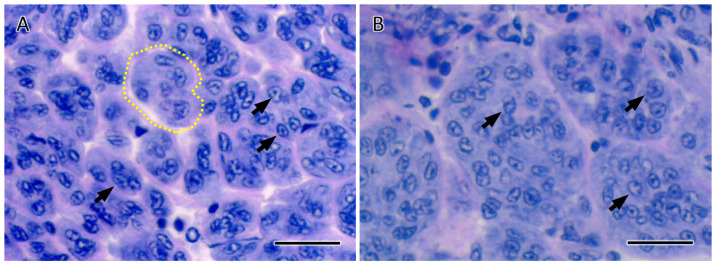
Histological images of the AG in Experiment 1. Representative sections of the AG on day 14 after ESA, stained with hematoxylin and eosin, are shown. Panel (**A**) corresponds to the control group, and panel (**B**) to the bilateral ESA group. Black arrows indicate AG cells, and the area enclosed by a yellow dotted line indicates a typical vesicle. Scale bar: 20 µm.

**Figure 4:**
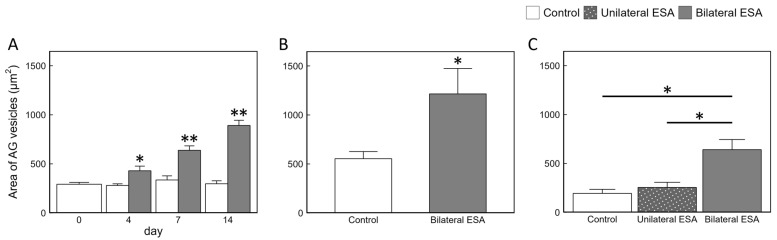
Quantitative analysis of AG vesicle area. AG vesicle areas from day 0 to day 14 after ESA in Experiment 1 and on day 14 in Experiments 2 and 3 are shown as mean + SEM. Sample sizes were as follows: (**A**) control group, *n* = 7, 5, 5, and 5 on days 0, 4, 7, and 14, respectively; bilateral ESA group, *n* = 3, 5, and 5 on days 4, 7, and 14, respectively; (**B**) both groups, *n* = 5, respectively; (**C**) control group, *n* = 5; unilateral ESA group, *n* = 5; bilateral ESA group, *n* = 5, respectively. Asterisks indicate significant differences on the same day, as determined by Wilcoxon’s rank-sum test or Steel-Dwass test (* *p* < 0.05, ** *p* < 0.01).

Histological observation was performed to examine whether ESA affected AG development and spermatogenesis. In this species, the AG consists of numerous vesicles attached externally to the SV (Figure S3), and AG cells are located within these vesicles. In Experiment 1, well-developed AGs were observed in both groups at all sampling days, and the AG vesicles in the bilateral ESA group were enlarged on day 14 (Figure 3). The vesicle area was measured at all sampling days. In the bilateral ESA group, the vesicle area was 1.5-, 1.9-, and 3.0-fold larger than that of the control group on days 4, 7, and 14, respectively (Figure 4A). Moreover, a significant monotonic increasing trend in vesicle area was detected in the bilateral ESA group during the experimental period. No abnormalities in the spermatogenesis, such as extreme bias in developmental stages in the testis and appearance of abnormally shaped sperm in the VDs and spermatophores, were observed in the bilateral ESA group (Figure S4).

In Experiments 2 and 3, the AG area was analyzed on day 14. In Experiment 2, well-developed AGs were also observed in the bilateral ESA group (Figure S5A,B), and the vesicle area was 2.2-fold larger than that in the control group (Figure 4B). In Experiment 3, the vesicle area in the bilateral ESA group was 2.5- and 3.3-fold larger than in the unilateral ESA and control groups, respectively (Figure S5C–E and Figure 4C). Enlargement of AG vesicles induced by bilateral ESA was consistently observed across all three experiments. AG vesicles in the control groups differed among experiments and tended to be larger in larger individuals: in Experiment 3, the mean AG vesicle area and BW were 193.6 ± 41.7 µm^2^ and 10.6 ± 0.4 g; in Experiment 1, 297.2 ± 30.5 µm^2^ and 21.9 ± 0.7 g; and in Experiment 2, 553.8 ± 73.1 µm^2^ and 29.6 ± 0.5 g, respectively (Figure 4).

## 4. Discussion

In this study, we present the first analysis of the effects of ESA on *Maj-IAG* expression together with changes in AG vesicle area and male reproductive organ indices (TSI, VDSI, SVSI) in *M. japonicus*. The consistent upregulation of *Maj-IAG* observed from day 7 onward across different growth stages and experimental conditions suggests that removal of eyestalk-derived inhibitory factor(s) drives sustained activation of the AG in this species. These results indicate that eyestalk regulation of IAG, widely reported among decapods, functions as a robust mechanism in *M. japonicus* across different growth stages and under varying physiological states after the onset of spermatogenesis. Changes in *IAG* expression after ESA have been reported in various decapod species [14,15,16,24,26,36,37]; however, the temporal patterns of *IAG* upregulation vary among species. For example, Chung et al. [14] reported that *IAG* expression in *C. sapidus* remained comparable to intact individuals at day 4 after ESA but increased significantly by day 7, a pattern similar to that observed in our study. In contrast, an earlier increase in *IAG* expression, as early as day 1 after ESA, has been reported in *F. merguiensis* [17]. Similar early upregulation was also reported in the swimming crab *Portunus trituberculatus*, the oriental river prawn *Macrobrachium nipponense*, and *M. rosenbergii*, with increases observed by day 2 or 4 [15,24,26,36]. Differences in these timings may be attributable to variations in experimental conditions, physiological states, and species-specific factors. To clarify the underlying causes, it would be desirable to conduct experiments on multiple closely related species under comparable conditions. In such experiments, including sampling time points earlier than day 4 would allow a more comprehensive understanding of the temporal profiles of IAG upregulation.

Unilateral ESA in Experiment 3 did not elevate *Maj-IAG* levels, which is inconsistent with previous findings [15,17,23,24,26]. Notably, the early increase in *IAG* expression from day 1 reported in *F. merguiensis* occurred following unilateral ESA [17]. In *M. nipponense*, *IAG* expression was significantly higher in the bilateral ESA group than in either the intact or unilateral ESA groups. Moreover, expression in the unilateral ESA group was significantly higher than in the intact group but significantly lower than in the bilateral ESA group on both day 4 and 7 [15]. Together with the interspecific variation in the timing of *IAG* upregulation after bilateral ESA described previously, these results suggest that, although regulation of *IAG* expression by eyestalk-derived inhibitory factor(s) is common among decapods, there may be interspecific differences in the relative contribution and strength of these inhibitory inputs. In *M. japonicus*, *Maj-IAG* suppression may normally be sufficiently maintained by inhibitory factor(s) released from a single eyestalk, or alternatively the remaining eyestalk may compensate for removal of the other by enhancing the production and secretion of the factor(s). By contrast, the available data suggest that such compensatory modulation of the eyestalk–AG axis may be weaker or absent in *M. nipponense*. These hypotheses could be tested in future studies by comparing the expression or secretion levels of the inhibitory factor(s) between the removed and remaining eyestalks. However, the identity of the central factor(s) that regulate *IAG* expression remains largely unknown. Recent studies in *L. vannamei* and *M. rosenbergii* have demonstrated that knockdown of specific CHH-family members expressed in the eyestalk significantly upregulates *IAG* expression [23,24]. These findings provide evidence that CHH-family functions as an upstream inhibitory regulator of *IAG* within the eyestalk–AG axis [24]. Further investigation into the relationship between IAG and eyestalk-derived factor(s), including CHH-family, will contribute to elucidating the overall regulatory mechanism mediated by the eyestalk. Another, not mutually exclusive, possibility is that the maturity state of males may have been an additional influencing factor. In mature female *M. japonicus*, unilateral ESA accelerates maturation and induces spawning [38], whereas in immature females, unilateral ESA does not induce maturation; only bilateral ESA promotes it [39]. A similar mechanism may play a role in regulating *Maj-IAG* expression.

In this study, *Maj-IAG* expression was significantly upregulated by bilateral ESA under all experimental conditions, whereas *Maj-Dsx1* showed no consistent ESA-induced increase. For *Maj-Dsx2*, an upward trend was observed at one or more time points in each experiment, but a statistically significant increase following ESA was detected only in Experiment 2. *Dsx* was first identified as polymorphic transcription factor in the fruit fly *Drosophila melanogaster*. In several insect species, sex-specific *Dsx* isoforms regulate sexual dimorphism [40,41,42,43,44,45]. By contrast, in the cladoceran crustacean *Daphnia magna* (water flea), two types of *Dsx* (*Dsx1* and *Dsx2*) have been identified, and *Dsx1* was demonstrated to act as a key regulator of the male phenotype through gene knockdown experiments [46]. In decapod crustaceans, *Dsx* has recently proposed as a candidate upstream regulator of IAG. In *M. rosenbergii*, *Dsx* expression in the AG increased following unilateral ESA [47]. Moreover, *Dsx* is considered to directly regulate *IAG* expression, based on the evidence of its binding to the IAG promoter and the reduction of *IAG* expression following *Dsx* knockdown in *M. rosenbergii, P. monodon*, and *E. sinensis* [27,28,29]. In addition, *Dsx* has been implicated in spermatogenesis in *E. sinensis* [27]. Collectively, these findings suggest that *Dsx* plays an essential role in sexual differentiation, development, and maturation across diverse Pancrustacea taxa, and that the decapod *Dsx* may regulate male sexual functions via transcriptional activation of *IAG* along the eyestalk–AG axis. Our comparisons of *Maj-Dsx1* and *Maj-Dsx2* expression patterns after ESA do not strongly support these previous reports; however, the significant ESA-induced increase in *Maj-Dsx2* in Experiment 2, along with similar but non-significant upward tendencies in Experiments 1 and 3, indicates that this gene remains a promising target for further investigation. As shown in Figure S3, the AG is a very small organ attached at the distal end of the SV, and while it may be possible to isolate the AG alone under a dissecting microscope, doing so reproducibly for a large number of individuals is not practically feasible. For this reason, we analyzed gene expression in the SV including the AG. Under these conditions, ESA-induced changes in gene expression within the AG may not have been detected with sufficient sensitivity, or the responses of non-AG tissues within the SV may have contributed substantially to the measured expression levels. Considering the nature of *Dsx* as a transcription factor, elucidating the direct relationship between *Dsx* and IAG will require more direct approaches, such as isolating the upstream region of *Maj-IAG* and performing promoter assays to test for *Dsx*-mediated transcriptional regulation. From the perspective of ESA responsiveness, it is noteworthy that the significant increase in *Maj-Dsx2* expression was observed only in Experiment 2, which used larger prawns and was conducted at relatively low water temperature. It is possible that, in addition to eyestalk-derived factors, *Maj-Dsx2* expression is modulated by other signaling pathways influenced by environmental conditions, such as temperature, and physiological states, such as body size.

In all three experiments conducted in *M. japonicus* at different growth stages, bilateral ESA resulted in a continuous increase in both TSI and VDSI (Figure 1). These increases suggest enhanced germ-cell proliferation and development in the testes, as well as sperm transport to VD, which were likely promoted by increased IAG secretion. A similar IAG-related stimulation of germ-cell proliferation has been reported in *M. rosenbergii* [48]. By contrast, in *C. quadricarinatus*, sperm duct weight (i.e., combined weight of VD and SV) relative to BW increased until week 2 and remained high through week 4, whereas testis weight relative to BW increased for two weeks but subsequently declined [19]. This transient increase has been interpreted as the prespermiation enhancement effect caused by ESA, possibly followed by feedback inhibition from the sperm duct [19]. A comparable pattern has also been observed in the coonstripe shrimp *Pandalus hypsinotus*, a species exhibiting seasonally fluctuating spermatogenesis [18]. In both *C. quadricarinatus* and *P. hypsinotus*, testicular weight increases before the reproductive season but decreases during the reproductive season [18,49], suggesting that the regulatory mechanisms linking spermatogenesis, sperm transport, and sperm storage differ between species that maintain mature sperm throughout the year (e.g., *M. japonicus*) and those with a limited mating period, including *C. quadricarinatus* and *P. hypsinotus*. Alternatively, extending the post-ESA observation period in *M. japonicus* (e.g., beyond 22 days) might reveal delayed testicular regression similar to that observed in these seasonally reproducing species.

Hypertrophy and hyperplasia of the AG induced by ESA have been reported in previous studies [18,19,20,21,48] and appear to represent a common phenomenon among decapods. Ultrastructural observations of AG cells in reproductively active *M. rosenbergii* revealed a well-developed rough endoplasmic reticulum (rER) in the cytoplasm, indicative of high protein synthesis activity. Following ESA, further ultrastructural changes have been described, including an increase in the number of rER, cytoplasmic hypertrophy filled with rER, and the development of granular vesicles [18,21], suggesting enhanced protein synthesis and AG cell enlargement [50]. These cellular changes are thought to contribute to hypertrophy and hyperplasia at the levels of AG cells, AG vesicles, and ultimately the entire AG. In the present study of *M. japonicus*, where the size of AG vesicles composed of AG cells was analyzed as an index, similar changes were observed. In Experiment 1, the area of AG vesicles began to enlarge by day 4 after bilateral ESA, with further hypertrophy observed on days 7 and 14 (Figure 4A). Together with the concomitant increase in *Maj-IAG* expression described above, these findings suggest that bilateral ESA induces functional activation of the AG in this species. In contrast, no significant change in AG vesicle area was observed after unilateral ESA (Figure 4C). Consistent with this result, the ultrastructure of AG cells in *P. hypsinotus* subjected to unilateral ESA was reported to be similar to that of intact individuals [18], suggesting that the remaining eyestalk compensates by producing and secreting greater amounts of inhibitory factor(s). The absence of increased *Maj-IAG* expression following unilateral ESA in *M. japonicus* supports the presence of a similar compensatory mechanism. Interestingly, the AG vesicle area of control prawns varied with body size in this study, and AG vesicles tended to be larger in larger individuals (Figure 4). However, the fold changes in *Maj-IAG* levels induced by ESA were almost the same regardless of differences in BW. Because the three experiments were conducted under different environmental conditions (e.g., water temperature and photoperiod), we cannot exclude the possibility that these factors also contributed to the variation in AG vesicle area. Nonetheless, these results suggest that, in prawns of the different body sizes after the onset of spermatogenesis, the secretory activity of the AG remains relatively constant, but the total secretory output increases as the AG vesicle area expands. In contrast, bilateral ESA induced significant increases in both AG vesicle area and *Maj-IAG* expression, indicating that bilateral ESA triggers a marked activation of AG function that exceeds the normal physiological range.

Our previous study showed that knockdown of *Maj-IAG* suppressed the development of the SV and VD but did not affect the testis [31]. Together, these results suggest that *Maj-IAG* primarily regulates the development of the SV and VD, whereas testis development after sexual differentiation may also depend on additional regulatory factors. Histological observation in the present study showed no clear differences in spermatogenesis within the testes between intact and eyestalk-ablated prawns (Figure S4A,B). Furthermore, no apparent morphological differences were observed between the two groups in the metamorphosed spermatozoa within spermatophores, capsule-like structures that transport sperm from males to females (Figure S4C–F). These observations are consistent with the results of our *Maj-IAG* knockdown study. Collectively, under the present experimental conditions, *Maj-IAG* does not appear to play a major role in regulating spermatogenesis in this species. Nevertheless, IAG is generally considered to be involved in the regulation of spermatogenesis in decapod crustaceans, and it will be important to carefully revisit whether *Maj-IAG* is truly unrelated to this process in *M. japonicus*. In the present study, ESA was performed on males after the onset of spermatogenesis, which may have masked any stimulatory effects on spermatogenic activity. It was also technically difficult to quantify sperm accumulated in the VD and SV because of the high viscosity of the contents. Given these limitations, future studies applying ESA at earlier developmental stages, prior to the onset of spermatogenesis, and establishing more accurate and standardized methods for evaluating spermatogenic function will be necessary to clarify the specific role of *Maj-IAG* in spermatogenesis. In addition, the initial VDSI and SVSI values among the three experiments tended to be higher in larger individuals (Figure 1), suggesting that the VD and SV were more developed in larger prawns. This pattern is in line with our previous finding that *Maj-IAG* is mainly involved in the development of the VD and SV rather than the testis [31], and with the interpretation from the present study that AG vesicle enlargement with increasing BW may reflect an increase in the total amount of IAG secreted, even if intrinsic secretory activity of the AG remains relatively constant.

Collectively, the removal of inhibitory factor(s) by ESA likely promoted the development of male reproductive organs through the upregulation of IAG. In our study, bilateral ESA induced *Maj-IAG* expression and enhanced the development of the testis, VD, and SV, whereas unilateral ESA had no such effect. These findings, together with our previous knockdown study [31], suggest that *Maj-IAG* expression level contributes to the extent of male reproductive organ development. Although the direct role of IAG in spermatogenesis remains uncertain, it has been proposed that it participates in the initiation and maintenance of spermatogenesis after reaching maturity [50]. Overall, our results support the presence of an eyestalk–AG–testis axis in *M. japonicus*, consistent with findings in other decapods, while AG-independent regulatory pathways cannot be excluded. Although many aspects remain unclear, including the possible involvement of *Maj-Dsx1* and -*Dsx2*, the present results strongly suggest that inhibitory factor(s) originating the eyestalk regulate *Maj-IAG* expression and that *Maj-IAG* functions as a principal mediator within the AG–testis endocrine axis.

## 5. Conclusions

This study clarified the regulatory mechanisms of *Maj-IAG* in the kuruma prawn *M. japonicus* by applying ESA. We demonstrated inhibitory regulation of *Maj-IAG* mediated by the eyestalk in individuals of different sizes. Bilateral ESA enhanced the activity of the AG, as evidenced by AG hypertrophy and increased *Maj-IAG* expression, thereby promoting the development of male reproductive organs. This finding supports the presence of an eyestalk–AG axis in male *M. japonicus*, as in other decapod crustaceans. Furthermore, our results showed that unilateral ESA did not induce a significant upregulation of *Maj-IAG* expression, nor did it promote the development of male reproductive organs. These results differ from previous reports in other species, suggesting that a complex regulatory mechanism might underlie the control of *IAG* expression in this species and that the relative contribution of eyestalk-derived inhibitory inputs to AG regulation may vary among decapods. Overall, this study provides fundamental insights into the regulation of male reproductive function in decapod crustaceans.

## Figures and Tables

**Table 1:** Detailed experimental conditions.

Experiments (Month)	Initial BW (g) ^a^	Water Temperature (°C)^ b^	Tank Condition	Light Condition ^c^	Treatment ^d^	Sampling Days
Exp. 1 (November)	22.6 ± 2.8	20.8 (19.1–22.4)	2-ton FRP tank in outdoor vinyl greenhouse	Covered with opaque light-shielding sheets (≥99% shading)	Bilateral ESA	0, 4, 7, 14
Exp. 2 (January~ February)	31.4 ± 1.9	14.1 (13.0–15.4)	Outdoor 6-ton reinforced concrete tank	Covered with light-shielding sheets (95~98% shading)	Bilateral ESA	0, 14, 22
Exp. 3 (November~ December)	8.78 ± 1.4	18.1 (16.0–19.1)	2-ton FRP tank in outdoor vinyl greenhouse	Covered with opaque light-shielding sheets (≥99% shading)	Bilateral and unilateral ESA	0, 7, 14

^a^ Values are shown as mean ± standard deviation. ^b^ Values were calculated from the temperatures recorded at 9:00 a.m. each day; values in parentheses indicate the range observed during the experimental period. ^c^ All experiments were conducted under natural daylength. ^d^ All experiments included control (intact) groups.

**Table 2:** List of the primers and probes used for RT-qPCR.

Name	Sequence (5′-3′)
Maj-IAG_fwd ^a^	CCTTGACCTGTTCCCTCAACA
Maj-IAG_rev ^a^	CCTTTCCTTTGTCCCTTCCAGAT
Maj-IAG_probe ^a^	CGCTTCCACCCTCGAGCCCTG
Maj-Dsx1_fwd ^b^	CATCAGCGATTACCCCCTTA
Maj-Dsx1_rev ^b^	CCCAGGGTTGTGTGAAGCTA
Maj-Dsx2_fwd ^b^	GGAGGCACCAGGATATGAAA
Maj-Dsx2_rev ^b^	GAGAGAAGCCTCCTGCGTAA

^a, b^ Sequences of primers and TaqMan probes are obtained from references Tsutsui et al. [33] and Toyota et al. [32], respectively.

## Data Availability

The original contributions presented in this study are included in the article/Supplementary Materials. Further inquiries can be directed to the corresponding author.

## Supplementary Materials

The following supporting information can be downloaded at https://www.mdpi.com/article/10.3390/ani15243556/s1, Figure S1: Initial body weight (BW) in each experiment; Figure S2: Body weight (BW) at each sampling day after eyestalk ablation (ESA); Figure S3: A cross section of the distal end of the seminal vesicle (SV) in a bilaterally eyestalk ablated prawn on day 14; Figure S4: Histological images of the testis, vas deferens (VD), and spermatophore on day 14 in Experiments 1; Figure S5: Histological images of the androgenic gland (AG) on day 14 in Experiments 2 and 3.
