# Supplementary material for: The Effects of Eyestalk Ablation on the Androgenic Gland and the Male Reproductive Organs in the Kuruma Prawn Marsupenaeus japonicus
Source: Animals (Basel). 2025 Dec 11;15(24):3556. doi: 10.3390/ani15243556 (PMC12729900; doi:10.3390/ani15243556)
Supplement: Supplementary file 1 [file animals-15-03556-s001.zip › Figure S1.pdf]

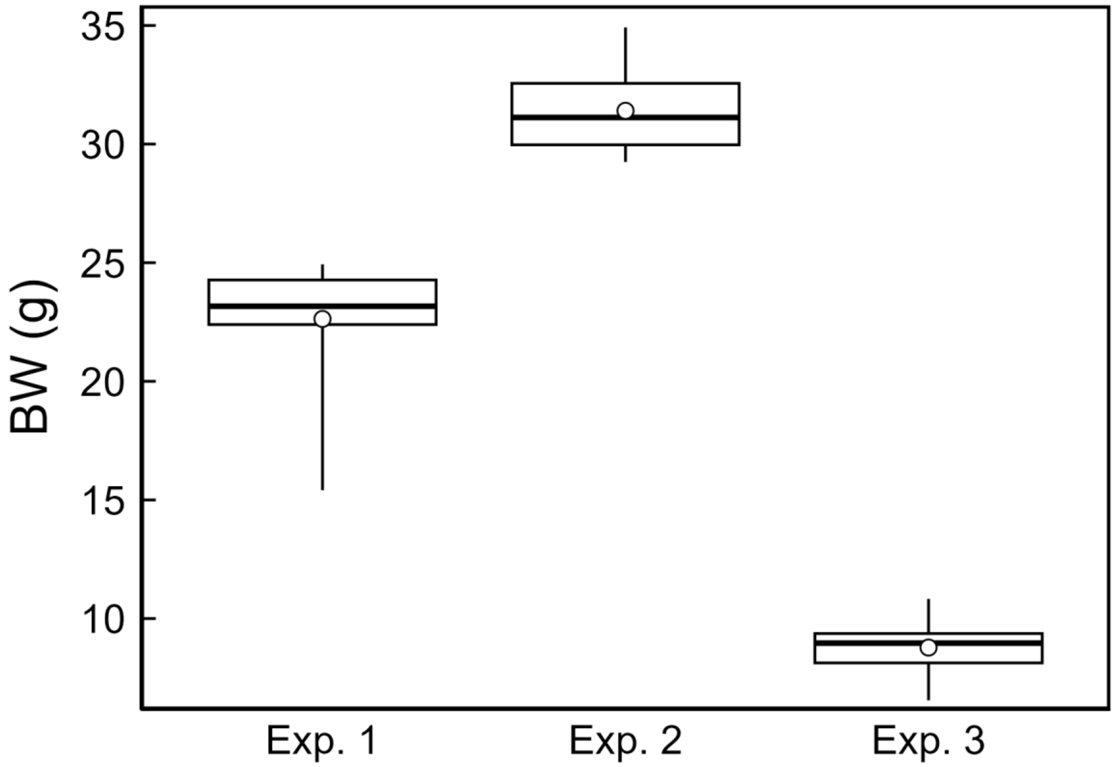

Figure S1. Initial body weight (BW) in each experiment. Boxplots show initial BW of the three experiments, with means indicated by white dots. Sample sizes were 10, 10, and 5 for Experiments 1–3, respectively. No outliers were removed for the construction of boxplots.
