# Supplementary material for: The Effects of Eyestalk Ablation on the Androgenic Gland and the Male Reproductive Organs in the Kuruma Prawn Marsupenaeus japonicus
Source: Animals (Basel). 2025 Dec 11;15(24):3556. doi: 10.3390/ani15243556 (PMC12729900; doi:10.3390/ani15243556)
Supplement: Supplementary file 1 [file animals-15-03556-s001.zip › Figure S2.pdf]

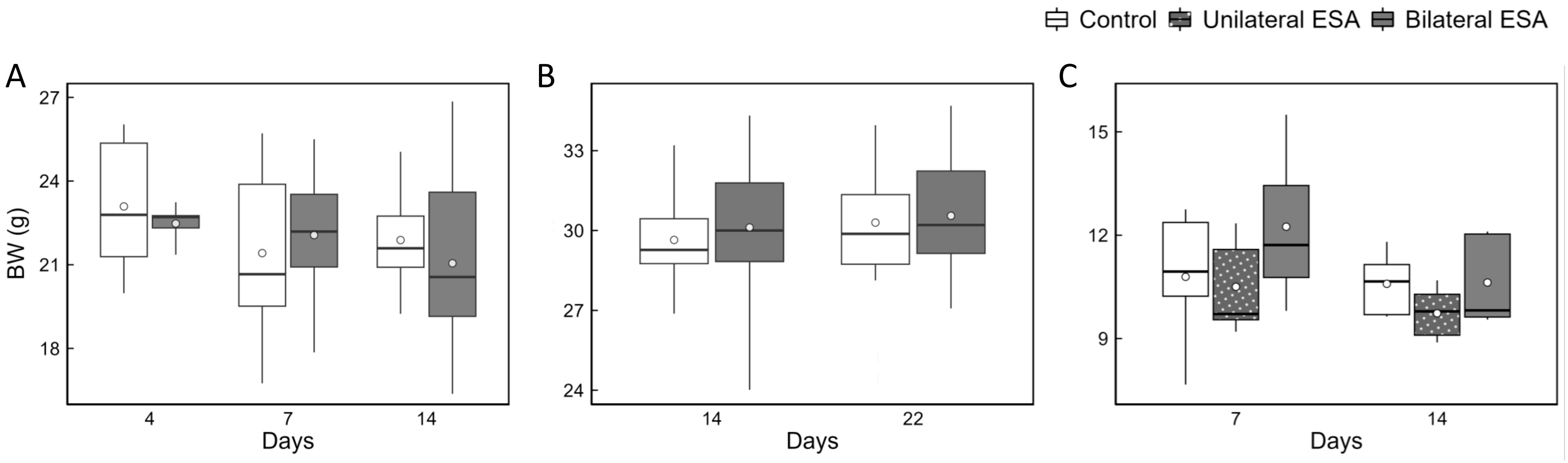


**Figure S2**. Body weight (BW) at each sampling day after eyestalk ablation (ESA). Boxplots show BW with means indicated by white dots. Sample sizes were as follows: (A) Experiment 1, control/bilateral ESA were 5/5 on day 4, 7/7 on day 7, and 7/9 on day 14; (B) Experiment 2, control/bilateral ESA were 12/15 on day 14 and 16/26 on day 22; (C) Experiment 3, control/unilateral/bilateral ESA were 5/7/5 on day 7 and 5/7/5 on day 14. No outliers were removed for the construction of boxplots. There were no significant differences between groups on the same day ($p > 0.05$, Wilcoxon's rank-sum test for Experiments 1 (A) and 2 (B), and Steel-Dwass test for Experiment 3 (C)).
