# Supplementary material for: The Effects of Eyestalk Ablation on the Androgenic Gland and the Male Reproductive Organs in the Kuruma Prawn Marsupenaeus japonicus
Source: Animals (Basel). 2025 Dec 11;15(24):3556. doi: 10.3390/ani15243556 (PMC12729900; doi:10.3390/ani15243556)
Supplement: Supplementary file 1 [file animals-15-03556-s001.zip › Figure S3.pdf]

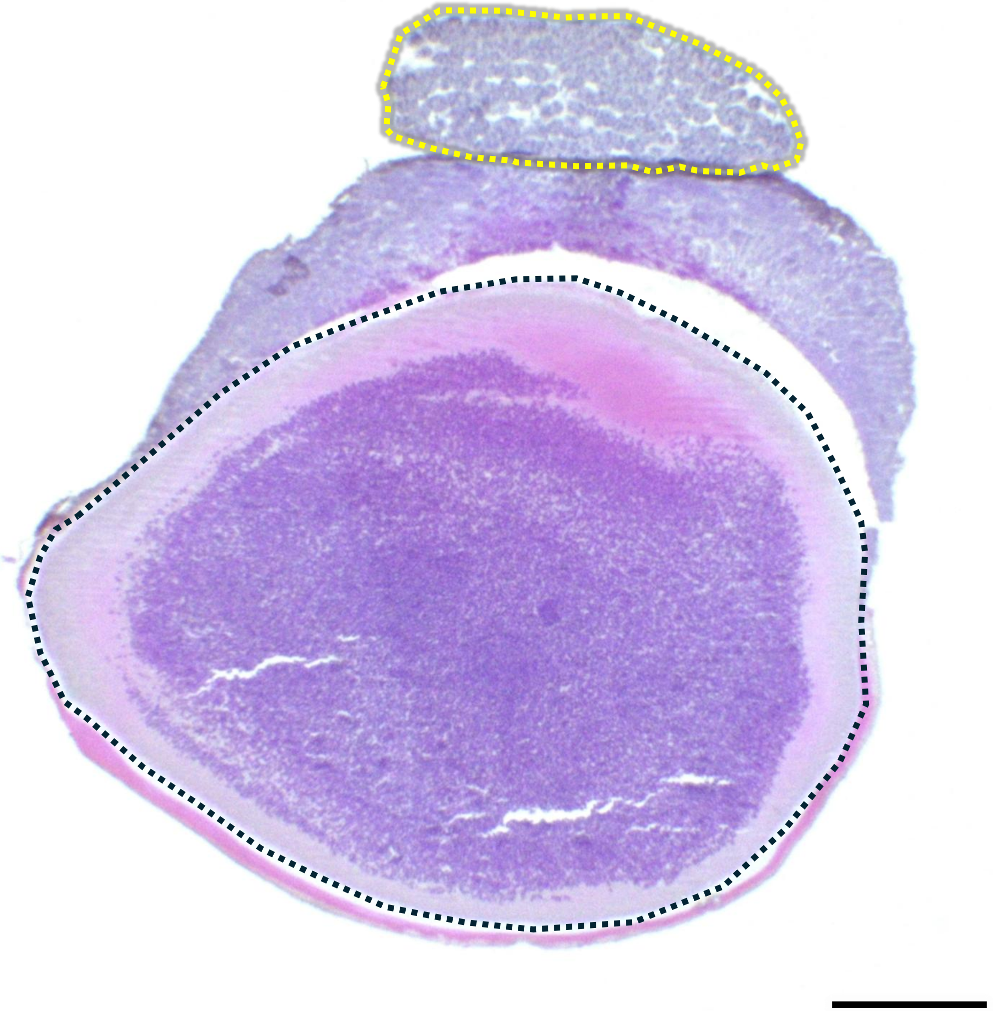

Figure S3. A cross section of the distal end of the seminal vesicle (SV) in a bilaterally eyestalk ablated prawn on day 14. The section was stained with hematoxylin and eosin. The androgenic gland and spermatophore are outlined by yellow and black dotted lines, respectively. Scale bar: 200 μm.
