# Supplementary material for: The Effects of Eyestalk Ablation on the Androgenic Gland and the Male Reproductive Organs in the Kuruma Prawn Marsupenaeus japonicus
Source: Animals (Basel). 2025 Dec 11;15(24):3556. doi: 10.3390/ani15243556 (PMC12729900; doi:10.3390/ani15243556)
Supplement: Supplementary file 1 [file animals-15-03556-s001.zip › Figure S4.pdf]

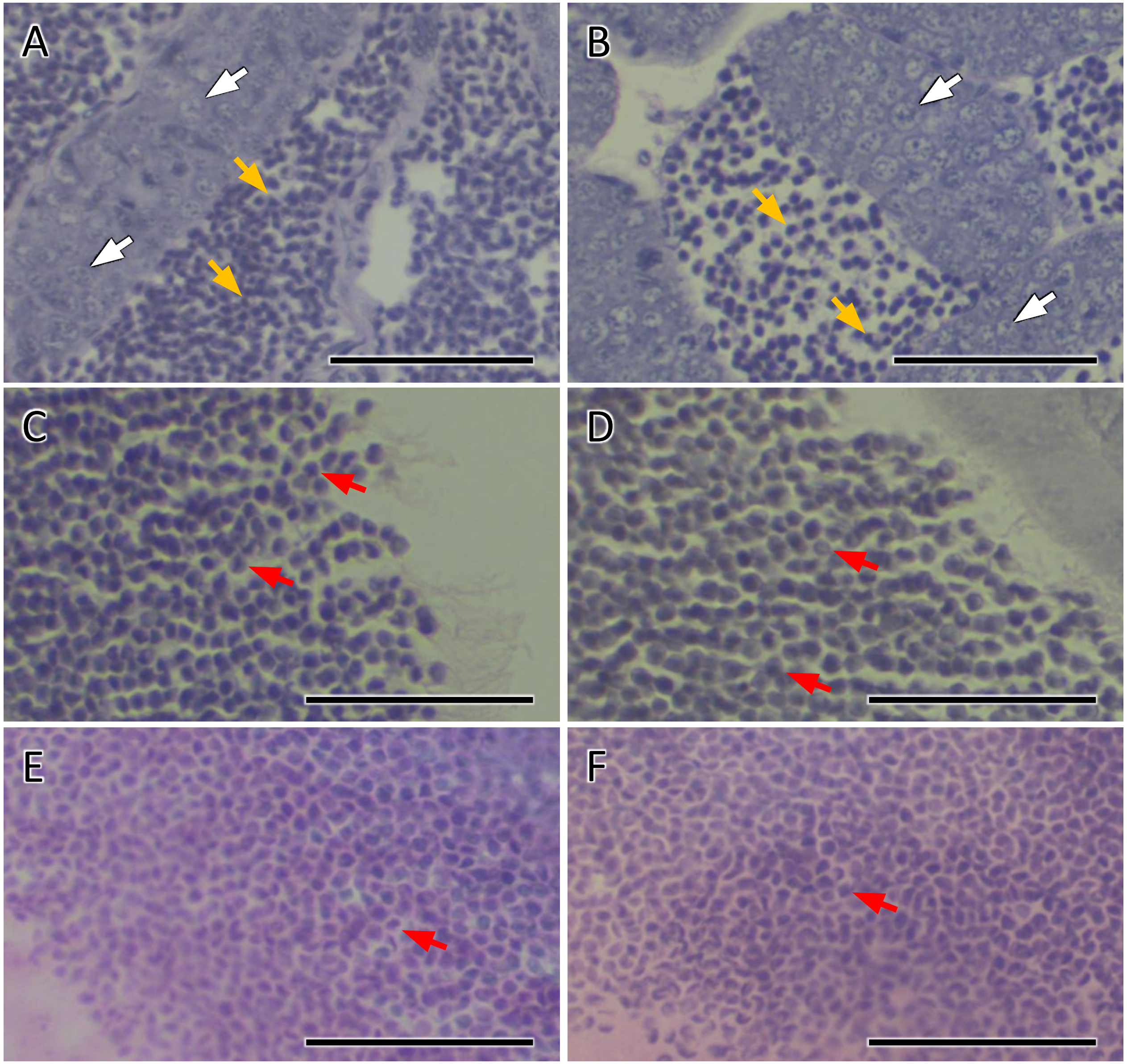

Figure S4. Histological images of the testis, vas deferens (VD), and spermatophore on day 14 in Experiments 1. The sections were stained with hematoxylin and eosin. Panels A, and B show representative sections of testis (control and bilateral ESA groups, respectively). Panels C and D show representative sections of VD (control and bilateral ESA groups, respectively). Panels E and F show representative sections of spermatophore (control and bilateral ESA groups, respectively). White, orange, and red arrows indicate spermatogonia, spermatid undergoing transformation into spermatozoa, and spermatozoa, respectively. Scale bars: 50 µm.
