# Supplementary material for: The Effects of Eyestalk Ablation on the Androgenic Gland and the Male Reproductive Organs in the Kuruma Prawn Marsupenaeus japonicus
Source: Animals (Basel). 2025 Dec 11;15(24):3556. doi: 10.3390/ani15243556 (PMC12729900; doi:10.3390/ani15243556)
Supplement: Supplementary file 1 [file animals-15-03556-s001.zip › Figure S5.pdf]

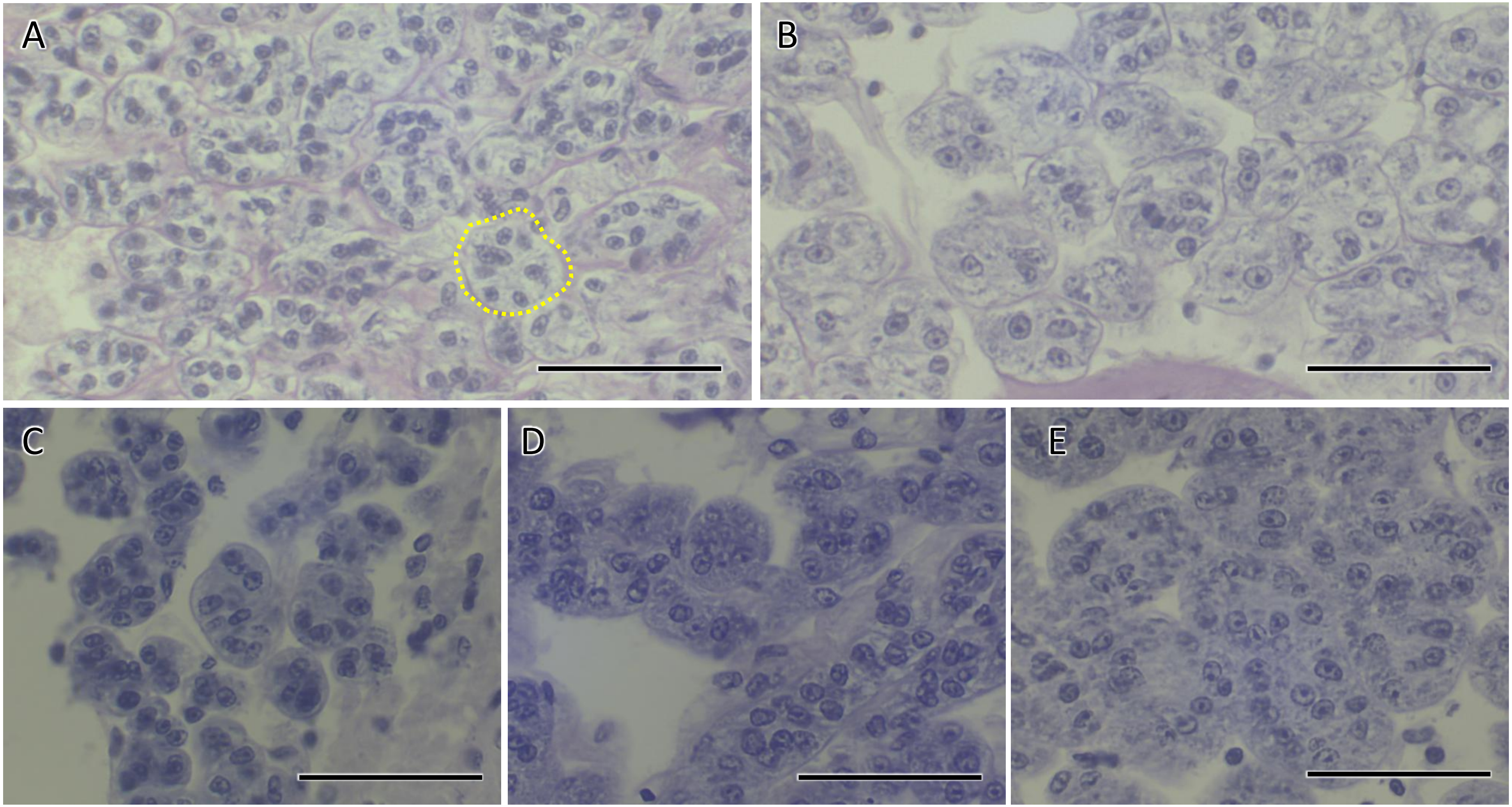

Figure S5. Histological images of the androgenic gland (AG) on day 14 in Experiments 2 and 3. The sections were stained with hematoxylin and eosin. Panels A and B show representative sections from Experiment 2 (control and bilateral ESA groups, respectively). Panels C, D, and E show representative sections from Experiment 3 (control, unilateral ESA, and bilateral ESA groups, respectively). A typical AG vesicle, composed of several AG cells, is outlined by yellow dotted line. Scale bars: 50 μm.
